# Appropriate Prescribing of Antipsychotic Medication for Non-Cognitive Symptoms in People With Dementia

**DOI:** 10.1192/bjo.2025.10621

**Published:** 2025-06-20

**Authors:** Huda Khan, Talha Rauf, Saba Inam, Odile Hally

**Affiliations:** North Dublin Mental Health Services, Dublin, Ireland

## Abstract

**Aims:** To evaluate current clinical practices in prescribing of antipsychotics in dementia patients for non-cognitive symptoms within our services and to assess whether antipsychotic medications are prescribed safely and reviewed appropriately.

**Methods:** 31 patients with a confirmed diagnosis of dementia who were prescribed antipsychotics for non-cognitive symptoms were selected. Patients with diagnosis of other co-morbid mental illnesses were excluded. Retrospective review of these 31 patients’ medical records was performed, where we looked for whether a comprehensive assessment was performed by a trained member of staff before prescribing antipsychotics, documented trial of non-pharmacological interventions before prescribing anti-psychotics, indications of anti-psychotic use, discussion about antipsychotics with the patients or caregivers before prescribing it, documentation of discussion made with patient or caregivers specifically mentioning risks and benefits including risk of stroke, TIA and mortality along with discussion regarding potential side effects including risk of falls, sedation and EPSEs, choice of antipsychotics, documentation of planned review after initiating antipsychotics, monitoring of side effects, outcome of planned review, consideration given to not use benzodiazepines for non-cognitive symptoms.

**Results:** All patients underwent a thorough evaluation by a trained healthcare professional before being prescribed antipsychotics. A documented trial of non-pharmacological interventions was conducted in 90.3%. Severe distress, accompanied by agitation, aggression, or psychosis, was observed in 51.6%. Additionally, 12.9% were at risk of self-harm, and 6.5% posed a risk to others. A documented discussion with the patient/or NOK regarding antipsychotics was held with 61.3%. And there was documentation of discussion with patient/or NOK regarding risks/befits specifically mentioning risk of stroke, TIA and mortality and side effects i.e. sedation, falls and EPSEs appeared in the medical record of 13 %. Regarding choice of antipsychotics risperidone was prescribed in 64.5% patients. Quetiapine was prescribed in 32.3% patients and olanzapine was prescribed in 3.2%. A review planned after initiating antipsychotics was documented in 96.8% patients, and all patients were monitored for side effects. Within three months of starting antipsychotic therapy, 61.3% patients had their dose increased, 22.6% patients continued the same dose, 12.9% patients had their dose reduced, and 3.2% patients the antipsychotic was discontinued. For 93.5% patients, benzodiazepines were intentionally avoided for managing non-cognitive symptoms.

**Conclusion:** The data highlights appropriate prescribing of antipsychotics medications within our services. Documentation regarding risk/benefits, specifically mentioning risk of stroke, TIA and mortality and discussion regarding the side effects of antipsychotic medication was not present in most cases, which can be a potential area for improvement.

